# Facile synthesis of hierarchical W_18_O_49_ microspheres by solvothermal method and their optical absorption properties

**DOI:** 10.1186/s11671-024-03990-1

**Published:** 2024-05-17

**Authors:** Yuanpeng Xiong, Bo Wu, Yuanzhi Lin, Mingwen Zhang, Jintian Chen

**Affiliations:** 1 School of Materials and Package Engineering, Fujian Polytechnic Normal University, Fuqing, 350300 China; 2 School of Materials Science and Engineering, Fuzhou University, Fuzhou, 350108 China

**Keywords:** Hierarchical structure, Nanowire, Optical absorption, Solvothermal synthesis, W_18_O_49_

## Abstract

In this study, a simple route for the synthesis of hierarchical W_18_O_49_ assembled by nanowires is reported. The morphologies and formation of W_18_O_49_ single-crystal could be controlled by changing the concentration of WCl_6_-ethanol solution. This synthesis strategy has the advantages that the hierarchical W_18_O_49_ microspheres could be economic synthesized at 180 °C without adding additives. Furthermore, efficient optical absorption properties in ultraviolet, visible and near-infrared region were obtained for the hierarchical W_18_O_49_ microspheres comparing with nanowires. These results will further promote the research of tungsten-based oxide nanomaterials.

**Graphical abstract:**

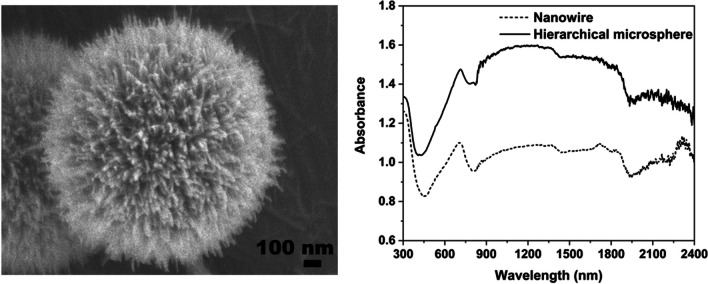

## Highlights

1. The hierarchical W_18_O_49_ microspheres assembled by nanowires were solvothermally synthesized at 180 ºC via using tungsten chloride and ethanol as reactants and without adding morphology control agent or using expensive alcohol, such as isopropanol or acetic acid.
2. The W_18_O_49_ nanowires tend to form hierarchical microspheres with the increase of WCl_6_-ethanol solution concentration.
3. The absorbance (300–2400 nm) of hierarchical W_18_O_49_ microspheres synthesized at 180 ºC were stronger than W_18_O_49_ nanowires synthesized at 200 ºC.

## Introduction

The synthesis of hierarchical structure assembled by low-dimensional nanostructures is a prerequisite for realizing the application of nanomaterials. The chemical and physical properties of hierarchical structures, as well as their applications, highly depend on their size, shape, and assembly of nanostructures [1, 2]. Many methods have been reported for the synthesis of hierarchical structures [3, 4]. However, it still remains a challenge to develop a facile, surfactant/stabilizer-free, economic synthesis strategy for the formation of hierarchical nanostructures.

Tugsten oxides-based nanostructure has been employed for a wide variety of applications due to its unique structure, such as gas sensors [5–8], electro-catalytic [9]. W_18_O_49_ is a kind of substoichiometric compounds, which contains tungsten ions of mixed valences along with the largest oxygen vacancy among the substoichiometric composition of tungsten oxide. The synthesis of W_18_O_49_ is of great interest due to its unusual structure. Nano-W_18_O_49_ has been found to have excellent optical absorption properties in near-infrared region [10–14]. The most common methods for the synthesis of hierarchical W_18_O_49_ nanostructures reported are limited to high temperature [15], using inorganic additives [16, 17], using expensive solvents, such as CH_3_COOH [18] or isopropanol [19].

Herein, we report a simple route for the synthesis of hierarchical W_18_O_49_ microspheres assembled by nanowires. The hierarchical W_18_O_49_ were solvothermally synthesized at 180 ºC using WCl_6_ and ethanol, and without adding morphology control agent.

## Experiment

### Materials

Tungsten chloride (99%) was purchased from Aladdin Co. Ltd. Ethanol was purchased from Beijing Chemical Co. Ltd. All reagents were analytical and used without further purification.

### Synthesis of W_18_O_49_

A certain amount of tungsten chloride was dissolved in 20 mL ethanol. Then, the volume of above solution was adjusted to 25 mL. The obtained solution was transferred into Teflon-lined autoclave of 50 mL internal volume, followed by solvothermal reaction in an electric oven under a certain temperature for 24 h. After the reaction, the obtained powder was centrifuged, washed 4 times with water and ethanol, and dried in a drying oven at 60 ºC.

### Characterization

The phase of samples was determined by X-ray diffraction (XRD, Rigaku D/max 2200 PC) using graphite monochromatized Cu Kα. The morphology and microstructure of samples were observed by scanning electron microscopy (SEM, JSM 7500F) and transmission electron microscopy (TEM, JEM-2100F).

### Optical test

The optical absorption properties were investigated by an ultraviolet–visible near-infrared spectrophotometer (UV–Vis-NIR, UV-3600) using BaSO_4_ as reference and equipped with integrating sphere. For UV–Vis-NIR, firstly, the absorbance of highly purified BaSO_4_ powder was used as baseline. Secondly, as-synthesized powder should be stuck to the surface of a sample stage, scraped and blown to form a layer as thin as possible, and its absorbance were tested via using the absorbance of BaSO_4_ powder as baseline.

The band gap (*E*_*g*_) of as-synthesized powder was calculated by employing their UV–Vis-NIR absorption spectra. The energy-dependent absorption coefficient *α* in the vicinity of the absorption edge can be expressed as Eq. (1) [15, 20, 21]:

1 $$\mathrm{\alpha h\nu }={\text{B}}{\left(h\nu -{E}_{g}\right)}^{n}$$

where B is a constant related the materials, *hν* is the incident photon energy, *n* depends on the kind of optical transitions. Specifically, *n* is 1/2 and 2 for transition being direct allowed and indirect allowed, respectively.

In accordance with Lambert–Beer’s law [21]:

$${\text{A}}=\mathrm{\alpha bc}$$

where A is absorbance, b and c are constants related the testing condition. For the samples, the value of b and c is certained. So, the equation can be described as α = A/bc = A/K. The eq. (1) can be given as follows:

2 $$\frac{Ah\nu }{KB}={\left(h\nu -{E}_{g}\right)}^{n}$$

## Results and discussion

### XRD analysis

**Fig. 1:**
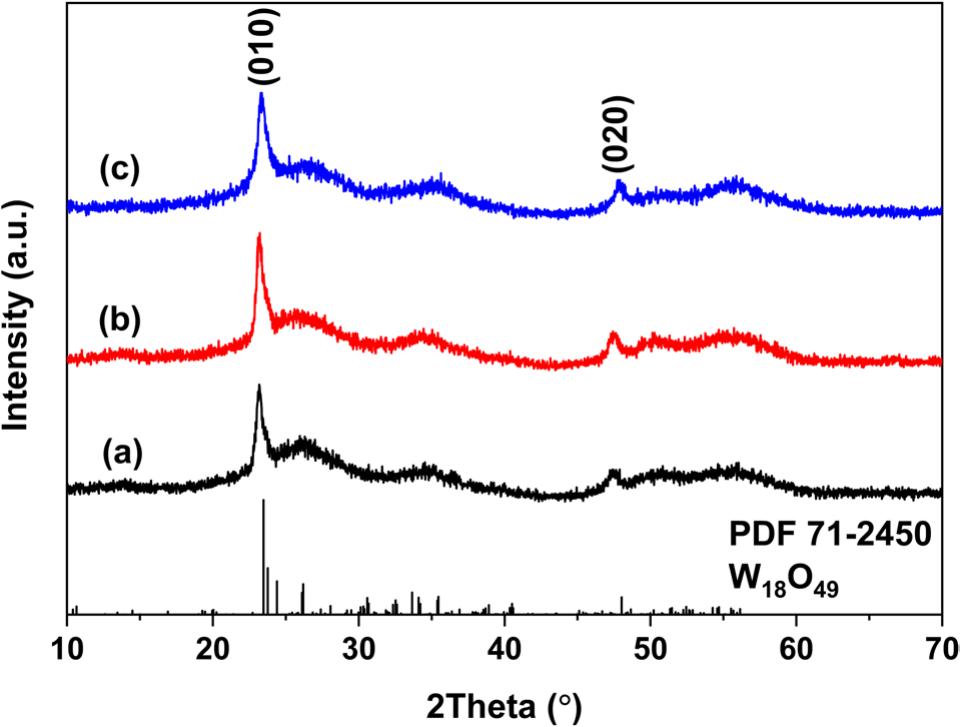
The XRD patterns of samples synthesized via **a** 0.005 mol/L; **b** 0.01 mol/L; **c** 0.02 mol/L WCl_6_-ethanol solution at 180 ºC

Figure 1 shows the XRD patterns of samples synthesized with different WCl_6_-ethanol concentration at 180 ºC. The diffraction peaks were similar with current reported literature [7]. All diffraction peaks were indexed to the monoclinic W_18_O_49_ (JCPDS No. 71-2450), and no impurity peaks were identified. It could be seen the (010) and (020) plane had the sharp diffraction peaks for all samples. Other peaks were weak and not sharp, indicating as-synthesized W_18_O_49_ tend to grow along [010] direction, which agree well with prior literature, since the close-packed planes of monoclinic W_18_O_49_ are (010) [17]. Noteworthy, the (010) and (020) peaks of samples synthesized with 0.005 mol/L and 0.01 mol/L WCl_6_-ethanol solution were slightly shift to lower angle. That is, as the WCl_6_-ethanol concentration was low, the mole fraction of oxygen vacancy in W_18_O_49_ was low because the ethanol is a source of oxygen in this route. The sample synthesized with 0.005 mol/L WCl_6_-ethanol solution had the lowest diffraction intensity, indicating it has the lowest crystallinity. As the WCl_6_-ethanol concentration was 0.02 mol/L, the diffraction peaks were matched well with standard card. The color of these samples was blue, which may originate from the chromophore of W^5+^ [22].

**Fig. 2:**
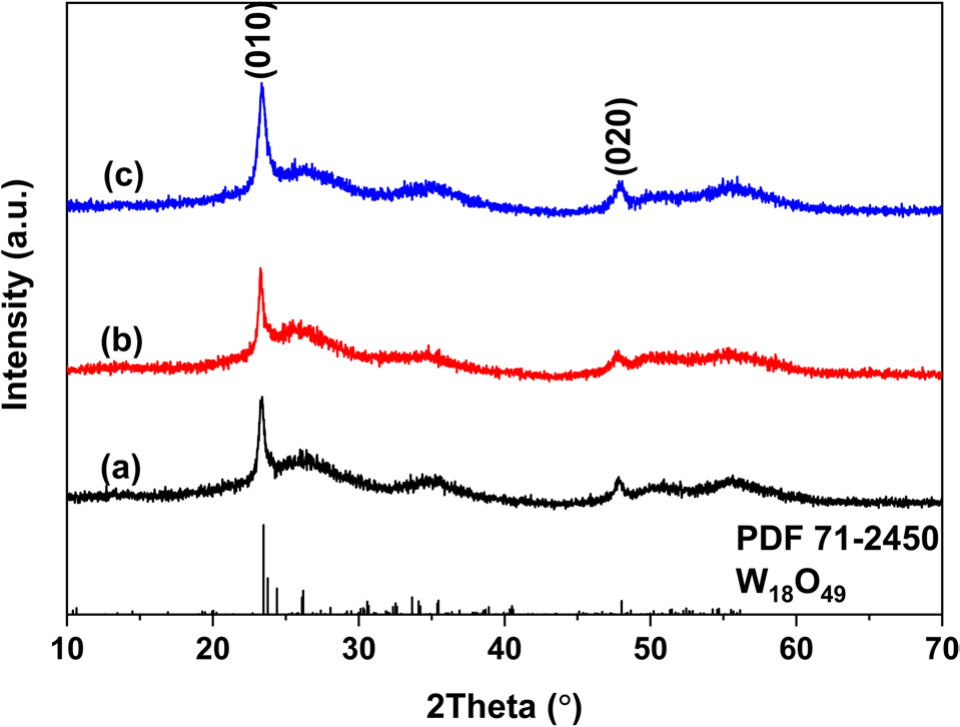
The XRD patterns of samples synthesized via **a** 0.005 mol/L; **b** 0.01 mol/L; **c** 0.02 mol/L WCl_6_-ethanol solution at 200 ºC

To further investigate the effect of reaction temperature on the phase, Fig. 2 shows the XRD patterns of samples synthesized with different WCl_6_-ethanol concentration at 200 ºC. Comparing with those synthesized at 180 ºC, the phase of as-synthesized samples was similar. All samples diffraction peaks matched well with the standard card. All other peaks were weaker and not sharp except (010) and (020) peaks, indicating as-sythesized W_18_O_49_ tend to grow along [010] direction. This was similar with those synthesized at 180 ºC. The sample synthesized with 0.02 mol/L WCl_6_-ethanol solution had the strongest diffraction intensity, indicating it has highest crystallinity. More remarkably, the color of these samples was deeper blue than those synthesized at 180 ºC, this may be related to the higher reduction ability of ethanol derived from higher reaction temperature.

### Morphology analysis

**Fig. 3:**
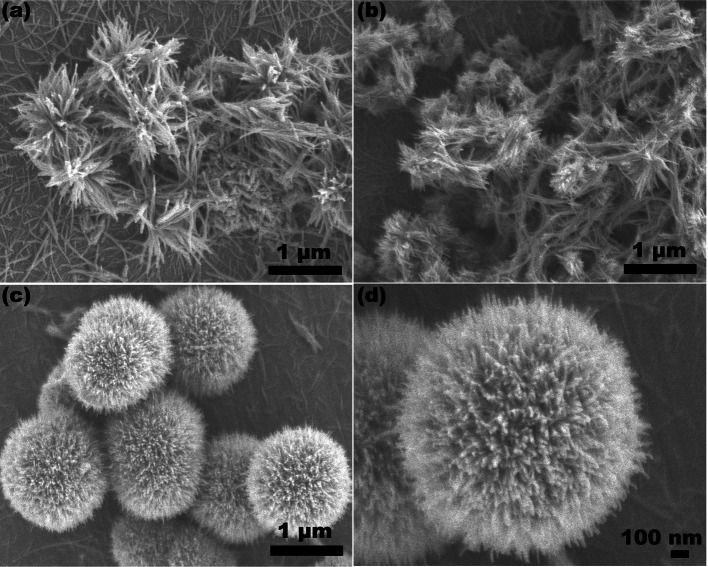
The SEM images of W_18_O_49_ synthesized via **a** 0.005 mol/L; **b** 0.01 mol/L; **c**, **d** 0.02 mol/L WCl_6_-ethanol solution at 180 ºC

Figure 3 shows the SEM images of W_18_O_49_ synthesized with different WCl_6_-ethanol concentration at 180 ºC. It could be seen the W_18_O_49_ synthesized via 0.005 mol/L WCl_6_-ethanol solution were consisted by nanowires (Fig. 3a). The length of nanowires exceeds 1 μm. As the WCl_6_-ethanol concentration was 0.01 mol/L, the W_18_O_49_ nanowires aggregate apparently (Fig. 3b). Some nanowires form a tightly bonded irregularity structure. As the concentration of WCl_6_-ethanol solution was 0.02 mol/L, the W_18_O_49_ were consisted by hierarchical microspheres with diameter around 1−2 μm (Fig. 3c). Further magnifying the SEM observation power, it could be seen the hierarchical microspheres were assembled by nanowires (Fig. 3d).

**Fig. 4:**
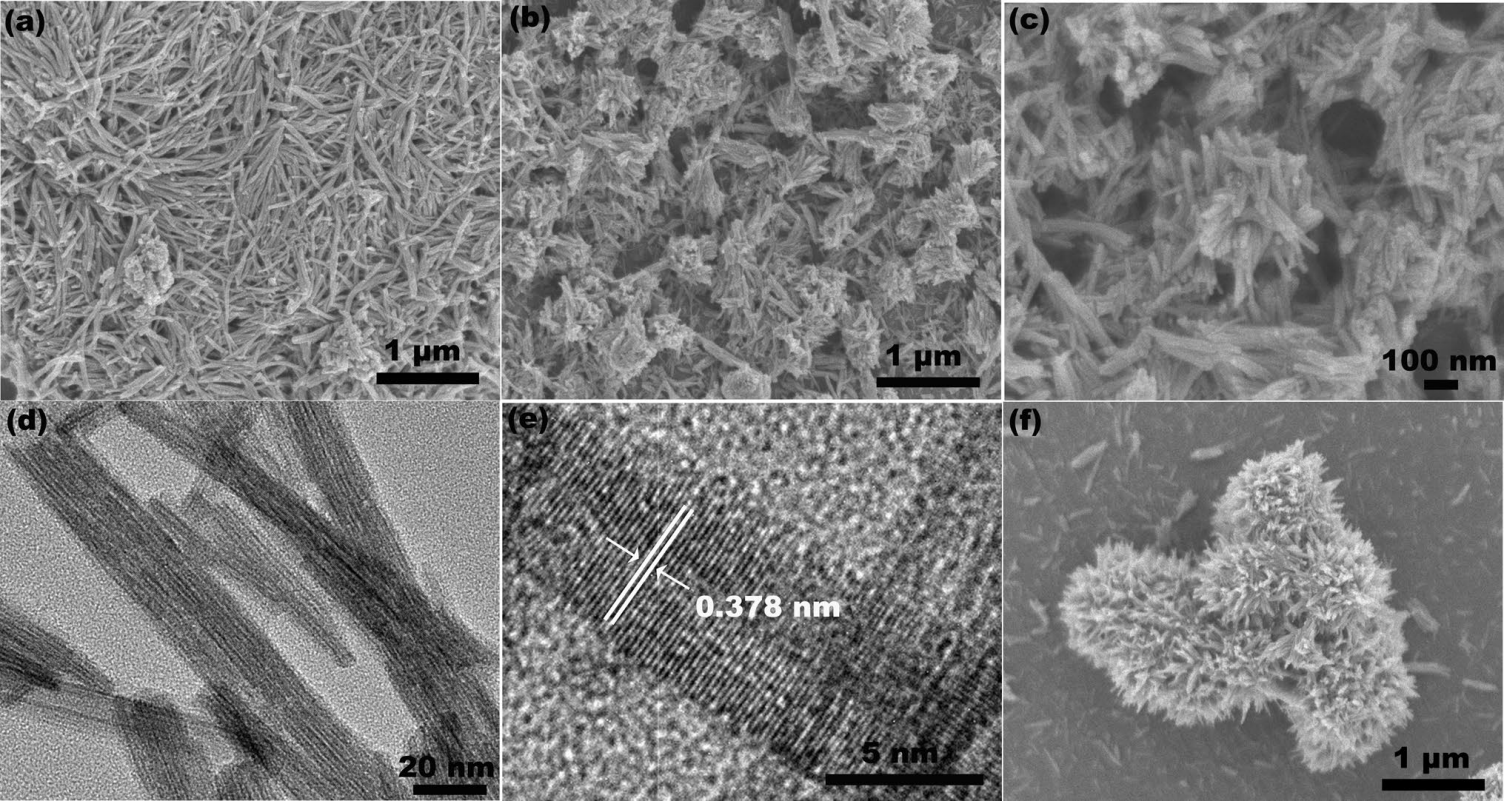
**a**, **b**, **c**, **f** SEM images and **d**, **e** TEM image of W_18_O_49_ synthesized via **a** 0.005 mol/L; **b**, **c**, **d**, **e** 0.01 mol/L; **f** 0.02 mol/L WCl_6_-ethanol solution at 200 ºC

Figure 4 shows the morphology of W_18_O_49_ synthesized with different WCl_6_-ethanol concentration at 200 ºC. It could be seen the W_18_O_49_ synthesized via 0.005 mol/L WCl_6_-ethanol solution were consisted by nanofibers with diameter around 20–30 nm and length exceed 1 μm (Fig. 4a). The diameter of nanofibers was larger than those synthesized at 180 ºC with similar WCl_6_-ethanol concentration, this may relate to the higher reaction temperature. As the concentration of WCl_6_-ethanol solution was 0.01 mol/L, W_18_O_49_ were consisted by some tightly bonded irregularity particles and randomly dispersed nanorods (Fig. 4b). Magnifying the SEM observation power, it was found that the irregularity particles were constructed by nanorods (Fig. 4c). Comparing with those synthesized via 0.005 mol/L WCl_6_-ethanol solution at 200 ºC, the length of nanorod were decreased to 0.3−1 μm. This may relate to the high WCl_6_-ethanol solution which contributed to high supersaturation of tungsten source, prohibited the growth of W_18_O_49_ along the [010] direction [23]. Further observed by TEM, it was found that the nanorods were assembled by nanowires (Fig. 4d). The HRTEM image of this sample show clear lattice fringes. The interplanar spacing of these fringes were 0.378 nm (Fig. 4e), agree with the (010) plane of the monoclinic W_18_O_49_. The TEM analytical results indicated that the W_18_O_49_ nanowires grow along [010] direction, which agree with XRD analysis. As the concentration of WCl_6_-ethanol solution was 0.02 mol/L, the morphology of W_18_O_49_ was transformed to hierarchical microspheres with diameter around 1–2 μm (Fig. 4f).

### Formation mechanism

**Fig. 5:**
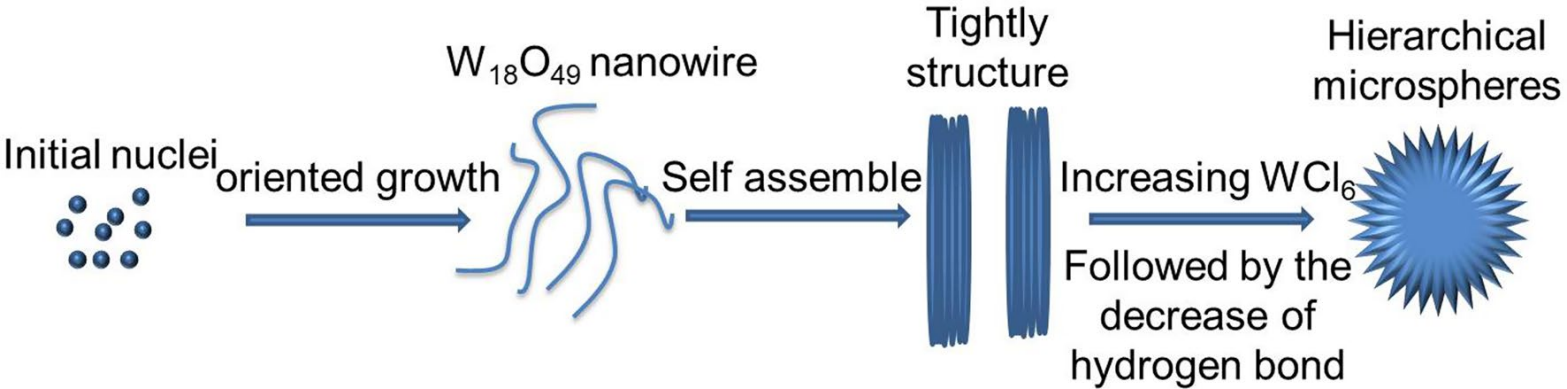
Schematic of the self-assembly processes to give a hierarchical structure of W_18_O_49_

Based on above results, it could be concluded that increasing the WCl_6_-ethanol concentration was benefit for the synthesis of hierarchical W_18_O_49_ microspheres. Figure 5 depicts a probable crystal growth process via self-assembly of W_18_O_49_. It could be divided into three steps, *i.e.*, firstly, a large number of W_18_O_49_ crystal nuclei were formed under solvothermal conditions. Since the close-packed planes of monoclinic W_18_O_49_ are (010), the crystal nuclei tend to grow along [010] direction and form W_18_O_49_ nanowires [16]. Secondly, it has been reported that oxygen vacancies exposed along the [010] direction in monoclinic W_18_O_49_ [24]. It led to the as-synthesized W_18_O_49_ nanowires have a high surface energy, and tend to aggregate to lessen the surface free energy, followed by forming a tightly bonded structure. Thirdly, with the increase of WCl_6_-ethanol concentration, as well as reaction time, the size of W_18_O_49_ nanowires increased, and the water produced by the reaction among ethanol molecules decreased, following the decrease of hydrogen bond. It led to the decrease of surface energy for the W_18_O_49_ nanowires, and weaken the bonding force among W_18_O_49_ nanowires. As a result, the tightly structure was transformed to hierarchical microspheres.

### Evaluation of optical absorption property

**Fig. 6:**
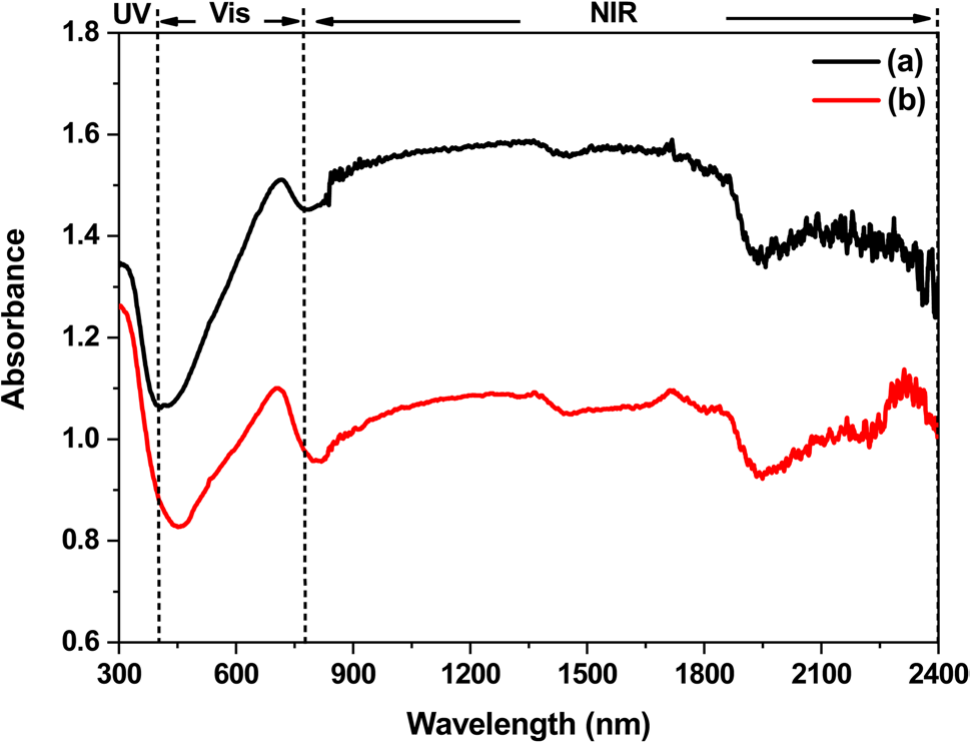
The UV–Vis-NIR spectra of **a** W_18_O_49_ hierarchical microspheres synthesized via 0.02 mol/L WCl_6_-ethanol solution at 180 ºC; **b** W_18_O_49_ nanowires synthesized via 0.01 mol/L WCl_6_-ethanol solution at 200 ºC

To investigate the optical absorption properties of as-synthesized W_18_O_49_ samples, Fig. 6 shows the UV–Vis-NIR spectra of W_18_O_49_ samples. In the region of 300–780 nm, it could be seen the absorbance of W_18_O_49_ hierarchical microspheres synthesized via 0.02 mol/L WCl_6_-ethanol solution at 180 ºC was stronger than W_18_O_49_ nanowires synthesized via 0.01 mol/L WCl_6_-ethanol solution at 200 ºC. The minimum absorbance for these samples occurs at about 450 nm (visible light region), indicating that both of them have a certain transmittance to visible light, and the hierarchical W_18_O_49_ microspheres have stronger ultraviolet absorbance than nanowires. Additionally, these samples have a main absorption around 700 nm. This may arise from the collective oscillations of excess electrons due to the existence of abundant oxygen vacancies in W_18_O_49_ [25]. The absorbance of hierarchical W_18_O_49_ microspheres around 700 nm is stronger than W_18_O_49_ nanowires. This may be attributed to the hierarchical structures, which facilitate the multiple light scattering between the radical nanowires [15].

**Fig. 7:**
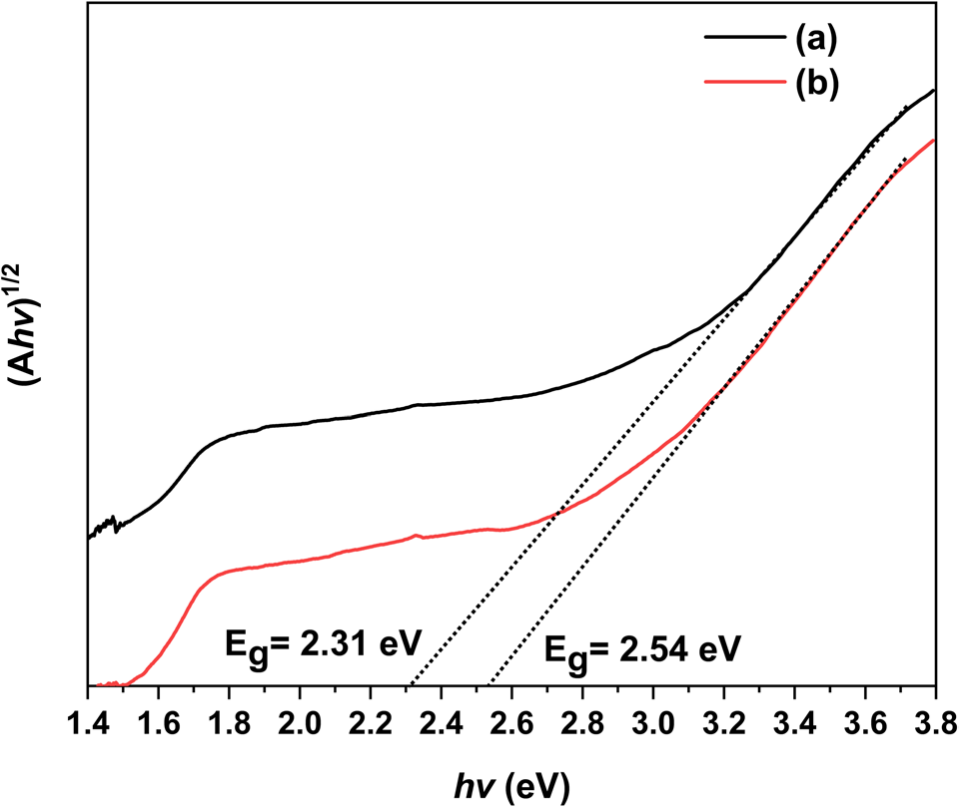
The band gap (*E*_*g*_) of **a** W_18_O_49_ hierarchical microspheres synthesized via 0.02 mol/L WCl_6_-ethanol solution at 180 ºC; **b** W_18_O_49_ nanowires synthesized via 0.01 mol/L WCl_6_-ethanol solution at 200 ºC calculated based on an indirect allowed transition ((A*hν*)^1/2^ vs.* hν*)

The W_18_O_49_ is an indirect semiconductor oxide [26], its absorption for ultraviolet and visible light may relate to band gap (*E*_*g*_). Figure 7 shows the band gap of as-synthesized W_18_O_49_ calculated based on an indirect allowed transition ((A*hν*)^1/2^ vs. *hν*). It could be seen the band gap of hierarchical W_18_O_49_ microspheres is 2.31 eV, which is smaller than W_18_O_49_ nanowires (*E*_*g*_ = 2.54 eV). This may be one of the reason resulted in the hierarchical W_18_O_49_ microspheres have stronger absorbance than W_18_O_49_ nanowires at 300–780 nm.

Additionally, in this study, ethanol not only acts as solvent, but also acts as reducing agent that transform the W^6+^ ions to W^5+^. The higher reaction temperature was benefit to induce more W^6+^ ions transform to W^5+^, following the increase of free electron concentration. However, the testing data show opposite results in Fig. 6, indicating the morphology of above samples play important role on their optical absorption properties.

In the near infrared region (NIR, 780–2400 nm), it could be seen that both of above W_18_O_49_ samples had broad absorption spectrum, and the hierarchical W_18_O_49_ microspheres had stronger absorbance than W_18_O_49_ nanowires. The properties of the hierarchical W_18_O_49_ microspheres should be good news for those who want to understand the near-infrared absorption of hierarchical tungsten oxides-based nanostructure constructed by low-dimensional nanostructures. Now, some researchers attribute the NIR absorption mechanism of tungsten-based oxide to many theories, such as plasmon resonance of free electrons [27, 28], small polaron [29], or interband transition [30]. These theories are somehow related to quantum confinement. For the one-dimensional nanostructure, the quantum confinement leads to the continuous energy level in one dimension transform to discrete energy level, the carriers in nanostructure cannot move free in this dimension. Under the radiation of near-infrared light, electrons in this dimension can be excited to high level, following the absorption of light. However, in the matter of hierarchical W_18_O_49_ structures responding to near-infrared light, how they work, is yet unknown. It was reported that the hierarchical nanostructure not only has the physical and chemical characteristics of low-dimensional nanostructures, but also can act as a mirror cavity [10], *i.e.*, the incident light enter into the cavity may be reflected many times, following the increasing number of absorption, thereby improve the NIR absorbance of hierarchical W_18_O_49_ microspheres. This may be one of the reason leads to hierarchical W_18_O_49_ microspheres have better NIR absorption. Moreover, smaller energy level spacing of hierarchical W_18_O_49_ microspheres (*E*_*g*_ = 2.31 eV) comparing with W_18_O_49_ nanowires (*E*_*g*_ = 2.54 eV) may be another reason, *i.e.*, it induces more electrons transit to higher energy level after absorbing photons, following improving of NIR absorption properties of hierarchical W_18_O_49_ microspheres [31, 32].

## Conclusions

In summary, hierarchical W_18_O_49_ microspheres assembled by nanowires were economic synthesized using a facile route based on self-assembly mechanism without additives. The WCl_6_-ethanol concentration play important role on the formation of hierarchical W_18_O_49_ structure, *i.e.*, W_18_O_49_ nanowires tend to form hierarchical structures by self-assembly of nanowires with the increase of WCl_6_-ethanol concentration. The XRD, SEM and TEM results confirmed the samples formation. In addition, the excellent optical absorption in the range of 300–2400 nm of hierarchical W_18_O_49_ microspheres was demonstrated. The morphology and band gap have significant effects on the optical absorption properties of W_18_O_49_.

## Data Availability

Data will be made available on request.
